# SMARCA4‐Deficient Undifferentiated Thoracic Tumor: Clinical Features and Prognosis of a Case Series and Literature Review

**DOI:** 10.1111/crj.70168

**Published:** 2026-01-23

**Authors:** Fangzhen Shan, Shuntao Liang, Youwen Zhang, Wei Wu, Guangxia Yang, Mei Yan, Xuemei Zhang, Yumao Miao, Linlin Liu, Jingjing Cai, Zhitao Shi, Bangdong Liu, Nannan Zhang

**Affiliations:** ^1^ Medical Research Center Affiliated Hospital of Jining Medical University Shandong China; ^2^ Biomedical Innovation Center Beijing Shijitan Hospital, Capital Medical University Beijing China; ^3^ Department of Pulmonary and Critical Care Medicine Affiliated Hospital of Jining Medical University Shandong China; ^4^ Department of Radiology Affiliated Hospital of Jining Medical University Shandong China; ^5^ Department of Pathology Affiliated Hospital of Jining Medical University Shandong China

**Keywords:** Brahma‐related gene 1, immunotherapy, SMARCA4, thoracic tumor, undifferentiated tumor

## Abstract

**Introduction:**

Thoracic SMARCA4‐deficient undifferentiated tumors (SMARCA4‐UT) are rare and aggressive epithelioid neoplasms characterized by the loss of the SMARCA4 gene. These tumors are typically diagnosed at advanced stages and exhibit a dismal prognosis. Currently, there are no standardized treatment protocols or approved targeted therapies.

**Methods:**

We present a case series of nine patients diagnosed of thoracic SMARCA4‐UT, detailing demographic, pathological, imaging, and treatment data. Moreover, a comprehensive literature review and genomic analysis of SMARCA4 mutations in lung cancer were also performed.

**Results:**

The cohort comprised predominantly male smokers (mean age: 63.0 ± 9.6 years). All cases exhibited loss of BRG1 expression, with negative staining for TTF‐1 and p40, while SMARCB1/INI‐1 expression was preserved. Patients showed poor responses to conventional chemotherapy but demonstrated partial responsiveness to immunotherapy or targeted agents. Genomic analysis of SMARCA4 mutations in lung cancer demonstrates that SMARCA4 mutations, primarily located in the SNF2‐related and helicase conserved C‐terminal domains, are associated with a poorer prognosis in lung cancer.

**Conclusion:**

Immunotherapy and targeted therapies show promise in managing thoracic SMARCA4‐UT, warranting further investigation. Further exploring the genetic and molecular landscape of this tumor might reveal potential therapeutic targets.

## Graphical abstract

Our manuscript presents detailed presentations of case series of thoracic SMARCA4‐UT, and we report a unique case of diaphragmatic metastases and, interestingly, the occurrence of brain metastasis in one patient, which is a rare phenomenon in SMARCA4‐UT. With a genetic analysis, it revealed that SMARCA4 mutations, primarily located within the SNF2‐related domain and the helicase conserved C‐terminal domain, are relatively common in lung cancer and are associated with a poorer prognosis.

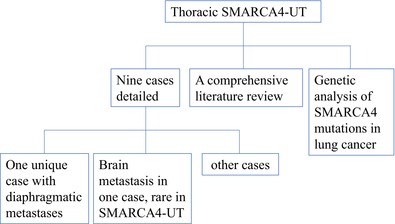

**Abbreviations**
ABCP: atezolizumab in combination with bevacizumab, paclitaxel, and carboplatin
ALK: anaplastic lymphoma kinase
CHD: coronary heart diseases
CI: cerebral infarction
CR: complete response
CT: computed tomography
DM: diabetes mellitus
EC: etoposide and carboplatin
EZH2: enhancer of zeste homolog 2
FLAIR: fluid‐attenuated inversion recovery
GP: gemcitabine and platinum
HBP: high blood pressure
HE: hematoxylin and eosin
ICI: immune checkpoint inhibitors
IHC: immunohistochemical
INI‐1: integrase interactor‐1
MRI: magnetic resonance imaging
NA: not available
NSCLC: nonsmall cell lung cancer
LN: lymph node
PC: paclitaxel and carboplatin
PD‐1: programmed death 1
PD‐L1: programmed death ligand 1
PR: partial response
RT: radiotherapy
SCLC: small cell lung cancer
SMARCA4‐UT: SMARCA4‐deficient undifferentiated tumor
TNM: tumor node metastasis
TPS: tumor proportion score
TTF‐1: thyroid transcription factor‐1

## 1 Introduction

Thoracic SMARCA4‐deficient undifferentiated tumors (SMARCA4‐UT) are rare, aggressive neoplasms that have recently been recognized as a distinct entity [1, 2]. These tumors, initially classified as SMARCA4‐deficient thoracic sarcomas or nonsmall cell lung carcinomas (NSCLC) [3, 4], are now formally recognized in the fifth edition of the WHO Classification of Thoracic Tumors [5]. Despite advancements in cancer therapy, effective treatments for SMARCA4‐UT remain elusive, with traditional cytotoxic agents showing limited efficacy [3]. The prognosis for SMARCA4‐UT is notably worse than for SMARCA4‐deficient NSCLC [1].

SMARCA4, a component of the mammalian switch/sucrose nonfermentable (SWI/SNF) chromatin remodeling complex, plays a critical role in gene transcription regulation [6]. Its loss disrupts normal cellular processes, leading to altered gene expression and dysregulation of pathways involved in cell growth, differentiation, and DNA repair. As a tumor suppressor, SMARCA4 is frequently altered in approximately 20% of human malignancies [7]. These tumors are characterized by the loss of the SMARCA4 gene, encoding the brahma‐related 1 (BRG1) protein, which is essential for SWI/SNF chromatin remodeling and gene transcription regulation [8]. SMARCA4 mutations are prevalent in various cancers, including lung, gastric, and breast carcinomas, and are associated with aggressive tumor behavior and poor outcomes [2].

Researchers believe SMARCA4 loss may disrupt normal cellular processes, leading to altered gene expression patterns and dysregulation of pathways involved in cell growth, differentiation, DNA repair, and tumor formation [9]. Approximately 10% of NSCLCs exhibit SMARCA4 deficiency, with mutations detected in 24% of lung cancer cell lines [10]. Moreover, about 25% of cases harbor mutations in at least one SWI/SNF complex gene, with 9% harboring SMARCA4 mutations among 3416 NSCLC patients [11]. SMARCA4‐deficient undifferentiated thoracic tumors primarily affect younger adults, predominantly males, often with a history of smoking [12]. These tumors are characterized by their aggressive behavior, rapid growth, propensity to metastasize, and resistance to treatment [9]. Histologically, they display undifferentiated features, lacking the typical cellular structure seen in other lung cancers, and are negative for epithelial markers such as epithelial membrane antigen (EMA) and pan‐cytokeratin (PCK), distinguishing them from SMARCA4‐NSCLC [5]. Because of their rarity and distinct genetic profile, accurate diagnosis of these tumors can be challenging. Typically diagnosed at an advanced stage, SMARCA4‐UT exhibit poor responses to conventional therapies, resulting in significantly worse prognoses compared to other lung cancer types, with a survival time of approximately 4–7 months [10]. With limited efficacy to standard NSCLC chemotherapy regimens, no established treatment protocols exist for SMARCA4‐UT to date.

Given the rarity of thoracic SMARCA4‐UT, this study presents a case series of nine patients with thoracic SMARCA4‐UT, providing detailed clinical, pathological, and treatment data. Additionally, we also conducted a comprehensive literature review and genomic analysis to better understand this rare and aggressive tumor type.

## 2 Materials and Methods

### 2.1 Cases Selection

Nine patients diagnosed with thoracic SMARCA4‐UT between March 2022 and August 2023 were retrospectively identified from Affiliated Hospital of Jining Medical College. Clinical data were extracted from electronic medical records, with the last follow‐up conducted in August 2025. Overall survival (OS) was defined as the duration from treatment initiation to death from any cause. Written informed consent was obtained from all patients or their next of kin, and the study was approved by the ethics committee of the Affiliated Hospital of Jining Medical University (No. 2024‐03‐C021). Because of some patients seeking treatment at other hospitals during follow‐up, comprehensive information for all patients could not be obtained.

### 2.2 Histology and Immunohistochemistry

Standard techniques were used for hematoxylin and eosin (HE) staining and Immunohistochemistry (IHC). Paraffin‐embedded blocks were sectioned, deparaffinized, and processed according to manufacturer instructions. Antibodies against SMARCA4/BRG1 (ZSGB‐Bio), TTF‐1 (Maixin Biotech), p40 (ZSGB‐Bio), Napsin A (Maixin Biotech), SMARCB1 (INI‐1) (ZSGB‐Bio), CK5&6, CK7 (ZSGB‐Bio), and Ki67 (Maixin Biotech) were utilized. For programmed death ligand 1 (PD‐L1) expression assessment, pathological sections were stained with PD‐L1 antibody (DaKo). PD‐L1 expression was assessed using the tumor proportion score (TPS), with positive expression defined as TPS ≥ 1%. TPS was calculated by dividing the number of PD‐L1 positive tumor cells by the total number of tumor cells and multiplying by 100. Positive expression cutoff values were defined as TPS < 1% for negative, TPS ≥ 1%–49% for positive expression, and TPS ≥ 50% for high expression levels. Histology slides were reviewed by two experienced pathologists.

### 2.3 Genomic Analysis of SMARCA4 Mutations in Lung Cancer

Genomic analysis of SMARCA4 mutations in lung cancer was conducted using data from the cBioPortal database (https://www.cbioportal.org/) [PMID: 23550210].

### 2.4 Statistical Analysis

Statistical analysis was performed using Graphpad Prism 7.0 software (San Diego, CA, USA) and SPSS 20.0 software (SPSS Inc., Chicago, IL, USA). The Kolmogorov–Smirnov test was used to assess data normality. OS was analyzed using Kaplan–Meier curves. Two independent sample *t* tests or nonparametric analysis was employed to compare measurement data between two groups as appropriate. Results are presented as mean ± standard deviation (SD) for normally distributed data and as median (interquartile range) for nonnormally distributed data. A significance level of *p* < 0.05 was considered statistically significant.

## 3 Results

### 3.1 Clinical Characteristics

The cohort included eight males and one female, with a mean age of 63.0 ± 9.6 years. Most patients were heavy smokers, and common symptoms included cough, sputum production, and chest pain. The clinical characteristics of the SD‐UT cases are summarized in Table 1. Computed tomography (CT) findings revealed that seven out of nine cases had pulmonary emphysema or both emphysema and bullae, with one case having no smoking history. Imaging revealed large, poorly demarcated masses in the mediastinum and lungs, with metastases to lymph nodes, bones, and other organs. Pathological evaluation samples were obtained from lung tissues and lymph node puncture biopsies. The primary tumor sites were distributed as follows: 2 (22.2%) in the left upper lobe, 1 in the left lower lobe, 1 in the lung mediastinum, 1 in the right middle and lower lobe; 2 in the upper lobe of the right lung, and 2 in the right lower lobe. Metastatic sites included lymph nodes (100%), bone (22.22%), brain (11.11%), adrenal (11.11%), liver (11.11%), and chest wall (11.11%). Tumor‐node‐metastasis (TNM) staging at diagnosis was available for six patients, mostly indicating stage IV tumors. Blood tumor marker levels were not significantly elevated, with CEA at 5.67 (2.61, 14.48) ng/mL, NSE at 17.24 (13.21, 37.66) ng/mL, and CYFRA21‐1 at 9.19 (4.79, 16.1) ng/mL.

**TABLE 1:** Clinical characteristics of thoracic SMARCA4‐UT patients.

Case no.	Age (y)/sex	Underlying diseases	Smoking (page year)	Symptoms at diagnosis	CT findings of nontumor lung	Biopsy type	Primary tumor site	Metastatic sites	TNM at diagnosis
Case 1	64/M	DM, CHD	80	Cough and sputum	Not obvious	Cervical lymph node biopsy	Left upper lobe	Axilla, LN	IV
Case 2	68/M	HBP, DM	40	Cough and fever	Emphysema	Endobronchial biopsy	Right middle and lower lobe	Mediastinum, diaphragm, LN, chest wall	IV
Case 3	68/M	DM, CHD, HBP, CI	100	Cough and sputum	Emphysema	Ultrasound‐guided lung puncture biopsy	Left upper lobe	Brain, adrenal, LN	IV
Case 4	42/M	No	5	Chest pain	Emphysema, bullae	Ultrasound‐guided lung puncture biopsy	Right upper lobe	LN, bone	IV
Case 5	55/F	No	—	Cough and chest tightness	Emphysema	Endobronchial biopsy	Mediastinum	LN	Unknown
Case 6	64/M	CI	30	Cough	Not obvious	Endobronchial biopsy	Right upper lobe	LN	Unknown
Case 7	72/M	No	16	Cough and sputum	Emphysema, bullae	Endobronchial biopsy	Right lower lobe	LN	Unknown
Case 8	61/M	HBP, CI	40	Cough and sputum	Emphysema, bullae	CT‐guided lung biopsy	Right lower lobe	Bone, liver	IV
Case 9	73/M	CI	40	Cough and hemoptysis	Emphysema	CT‐guided lung biopsy	Left lower lobe	LN	III

Abbreviations: Chemo, chemotherapy; CHD, coronary heart diseases; CI, cerebral infarction; CT, computed tomography; DM, diabetes mellitus; F, female; HBP, high blood pressure; LN, lymph node; M, male; NA, not available; TNM, tumor node metastasis; SMARCA4‐UT, SMARCA4‐deficient undifferentiated tumor.

### 3.2 Imaging Features

**FIGURE 1:**
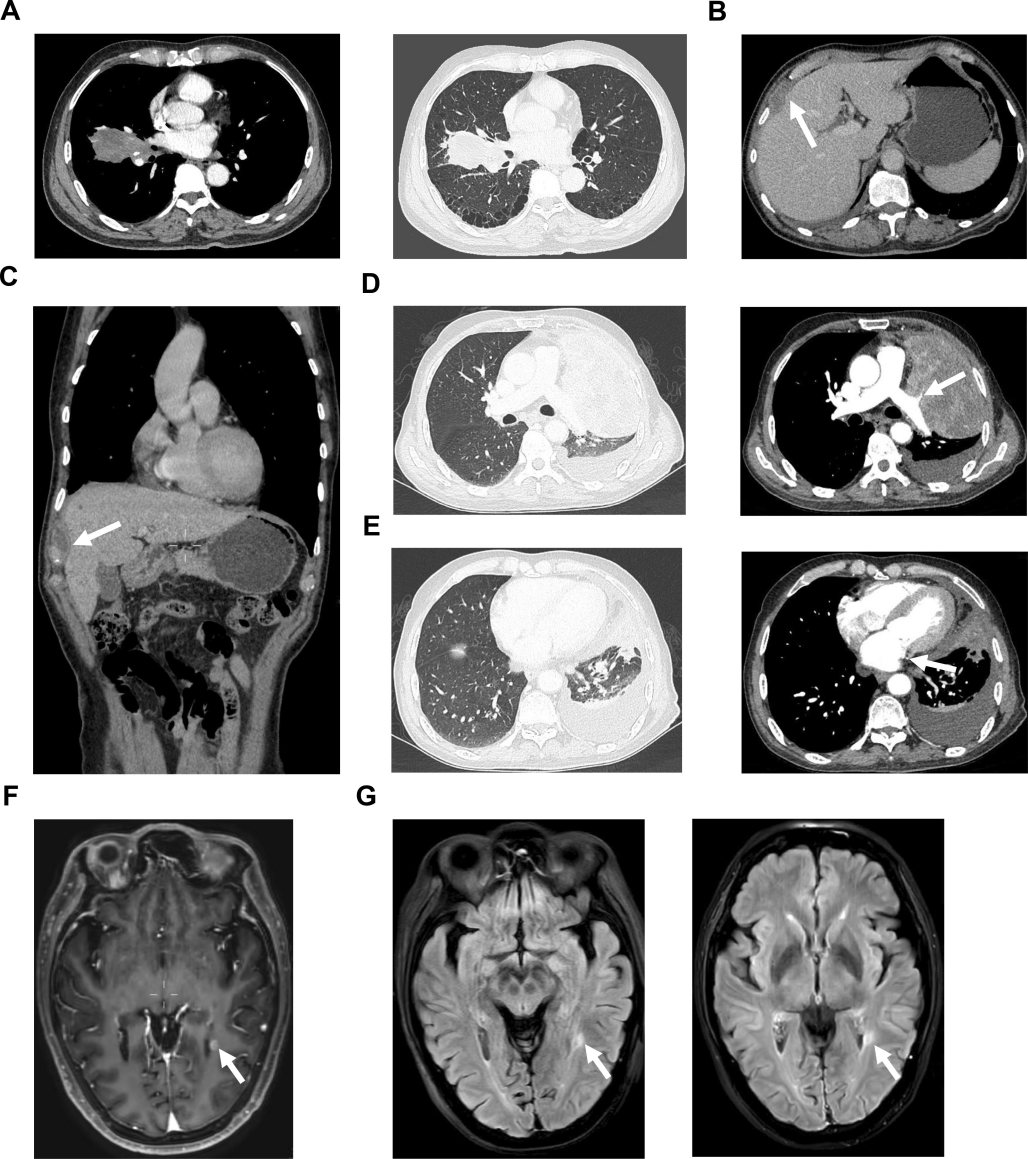
Images revealing diaphragmatic metastasis of Case 2 and brain metastasis of Case 3. (A) Chest CT at diagnosis of Case 2. (B) Low density observed in the diaphragmatic muscle at the right margin of the liver in Case 2. (C) Coronal view displaying a low‐density shadow in the diaphragm of Case 2 detected by chest CT. (D) Pulmonary occupation with invasion of pulmonary vessels indicated by chest CT at diagnosis of Case 3. (E) Involvement of the left atrium shown on chest CT of Case 3. (F–G) T1‐weighted signal mass (F) and T2‐weighted signal mass based on FLAIR images (G) indicated nodular enhancement in the brain parenchyma around the trigone of the left lateral ventricle in Case 3, suggesting the presence of metastatic tumor. Arrows indicate masses. CT, computed tomography; FLAIR, fluid‐attenuated inversion recovery; MRI, magnetic resonance imaging.

Chest CT images often showed a compressed, poorly demarcated mass, commonly located in the mediastinum and lung, with invasion into the pulmonary hilum, mediastinum, and distant lymph nodes. Notably, Case 2, with a smoking history of 40 pack‐years, had a CEA level of 1079.48 ng/mL. Imaging findings revealed a soft tissue mass in the middle and lower lobe of the right lung (7.3 × 3.7 × 3.4 cm), with metastasis in the right chest wall (Figure 1A). Additionally, hyperechoic nodules in the right anterior lobe of the liver were suggestive of diaphragmatic metastases (Figure 1B,C). Case 3 showed left upper lobe occupation with obstructive atelectasis, involvement of the left superior lobe pulmonary vascular, and invasion of the left atrium (Figure 1D,E). Brain metastasis, though rare in SMARCA4‐UT, was observed in one patient [13]. Magnetic resonance imaging (MRI) of the brain revealed a metastasis in the brain parenchyma around the trigone of the left lateral ventricle with a diameter of 4 mm (Figure 1F,G). Other cases demonstrated lung or mediastinum occupations with larger spaces, along with various metastatic sites. For Case 8, chest CT revealed a mass in the lower lobe of the right lung, a low‐density shadow on the liver margin, bone destruction in the left mandible, and the presence of right lung carcinoma with liver and bone metastases. Additionally, a head MRI showed metastasis to the left mandible.

### 3.3 Pathological Features

**FIGURE 2:**
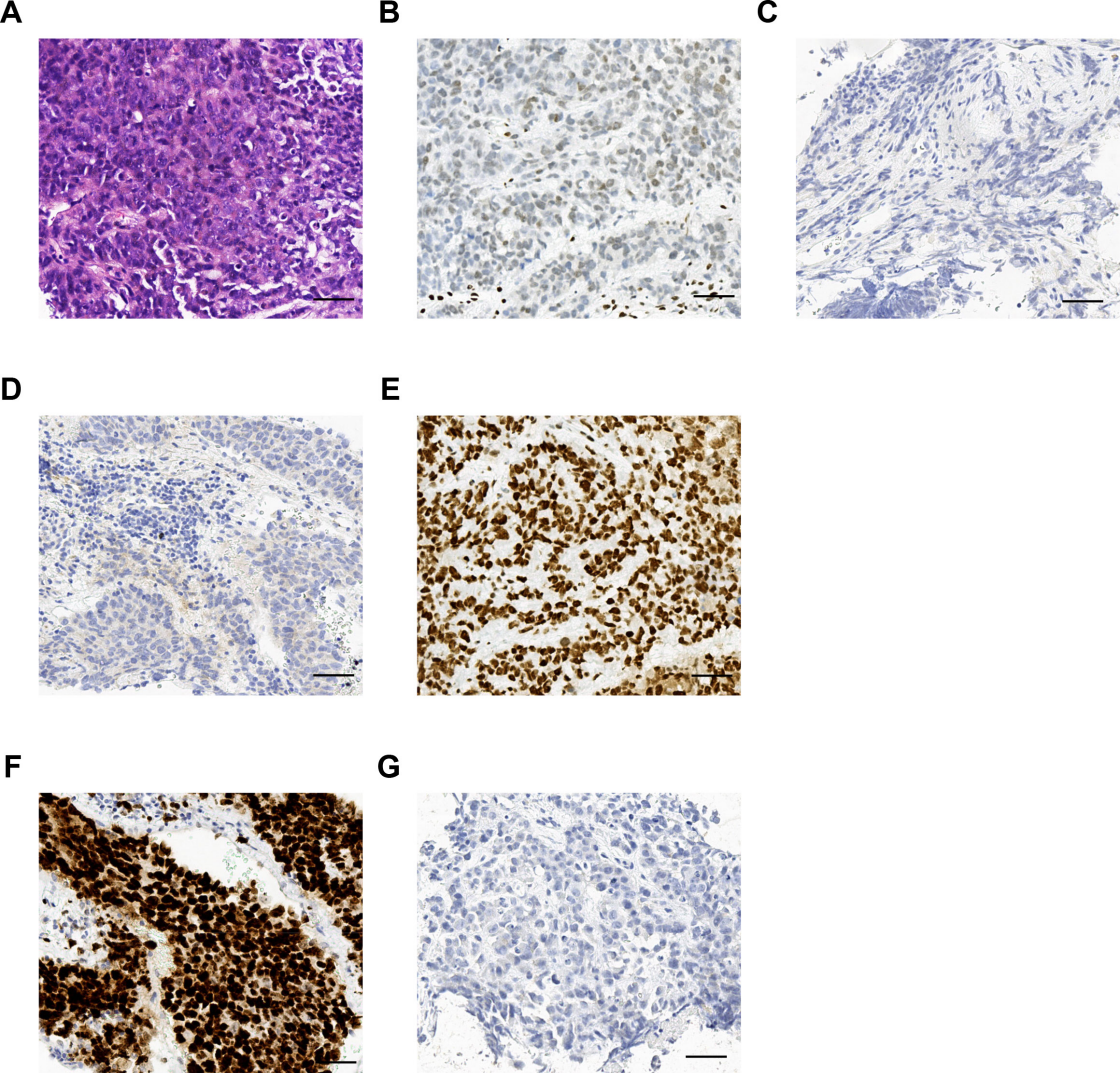
Morphologic and immunohistochemical features of SMARCA4‐undifferentiated thoracic tumors in Case 1. (A) HE staining revealing a poorly differentiated neoplasm with marked nuclear atypia, prominent nucleoli, pleomorphism, and rhabdoid cells. (B) IHC stain demonstrating complete loss of BRG1 in tumor cells. (C) Negative staining for TTF‐1. (D) Loss of p40 expression. (E) Preserved expression of SMARCB1 (INI‐1). (F) High Ki‐67 index (> 90%) detected by IHC staining. (G) Absence of PD‐L1 expression. Original magnification: × 400. HE, hematoxylin and eosin; IHC, immunohistochemical; INI‐1, integrase interactor‐1; PD‐L1, programmed death ligand 1; TTF‐1, thyroid transcription factor‐1.

The pathological features are detailed in Table 2 and Figure 2. SMARCA4‐UT specimens exhibit malignant undifferentiated cells with high pleomorphism, abundant cytoplasm, large nuclei, and a diffuse growth pattern, as depicted in Figure 2A. HE staining revealed poorly differentiated cells with marked nuclear atypia, prominent nucleoli, pleomorphism, and occasional rhabdoid cell morphology. Pathological characteristics of the cases are presented in Table 2, with complete absence of SMARCA4/BRG1 in all patients (Table 3, Figure 2B). Immunohistochemical analysis indicated negativity for lung adenocarcinoma markers TTF‐1 and squamous cell carcinoma markers p40, with retained INI‐1 expression (Figure 2C–E). Napsin A, a lung adenocarcinoma marker, was negative in all cases except for two unavailable cases (77.78%). CK7 expression was observed in one case and focally in three cases (44.44%). CK5/6 expression was negative in six cases, focally positive in two cases, and positive in one case (66.67%). Ki‐67 indices ranged from 15% to more than 90%, indicating high proliferative activity (Figure 2F). PD‐L1 detection was performed in five cases. The tumor cells lack of PD‐L1 revealed by IHC results of Case 1 (Figure 2G). Gene mutation examination was conducted for five patients (55.56%) and was only available for four patients (44.44%). Gene mutation analysis revealed KRAS alteration in Case 2 and an anaplastic lymphoma kinase (ALK) mutation in Case 7. The aggressive nature and widespread metastasis observed align with the diagnosis of SMARCA4‐UT, which is characterized by a lack of specific differentiation markers. In the series, TTF‐1, p40, and Napsin A were negative, while SMARCB1 (INI‐1) was preserved in all patients.

**TABLE 2:** Clinicopathologic and immunohistochemical profiles.

Case no.	BRG1	TTF‐1	P40	Napsin A	SMARCB1 (INI‐1)	CK7	CK5/6	Ki67 (%)	PD‐L1 (%)	Classical gene mutation
Case 1	−	−	−	−	+	−	−	> 90	< 1	No
Case 2	−	−	−	−	+	Focally+	−	> 75	< 1	KRAS
Case 3	−	−	−	NA	+	−	−	30–40	Untest	Untest
Case 4	−	−	−	NA	+	−	−	60–70	Unknown	No
Case 5	−	−	−	−	+	Focally+	Focally+	50	Untest	Untest
Case 6	−	−	−	−	+	+	−	15	Untest	NA
Case 7	−	−	−	−	+	−	Focally+	50–60	50	ALK
Case 8	−	−	−	−	+	Focally+	+	40–50	< 1	Undo
Case 9	−	−	−	−	+	−	−	60–70	Untest	Untest

Abbreviations: ALK, anaplastic lymphoma kinase; INI‐1, integrase interactor‐1; NA, not available; PD‐L1, programmed death ligand 1; SMARCA4‐UT, SMARCA4‐deficient undifferentiated tumor; TTF‐1, thyroid transcription factor‐1.

**TABLE 3:** Treatment and prognosis of the patients.

Case no.	ICI	Chemotherapy	Anti‐angiogenesis	Targeted drug	RT	OS (months)
Case 1	Tirellizumab	PC	Bevacizumab/recombinant human endostatin	—	—	4
Case 2	Atezolizumab/sint^c^	PC^a^; GP^b^	Anlotinib (third‐line)	—	—	7
Case 3	Triplimab	—	—	—	—	Extended for 38 months, alive until August, 2025
Case 4	Carelizumab	PC	—	—	Thorax	15
Case 5	Clinical trial (undisclosed)^d^	NA	—	—	—	34
Case 6	—	NA	—	NA	—	14
Case 7	—	—	—	Ensartinib	—	3
Case 8	—	GP	Recombinant human endostatin	—	—	8
Case 9	—	—	—	—	—	5

Abbreviations: GP, gemcitabine and platinum; ICI, immune checkpoint inhibitor; PC, paclitaxel and carboplatin; RT, radiotherapy.

^a^ Represents first‐line therapy.

^b^ Represents second‐line therapy.

^c^ Case 2 subsequently changed the immunotherapy therapy to sintilimab for economic reasons.

^d^ The patient was enrolled in the clinical trial, so the specific drug was unknown.

### 3.4 Treatments and Prognosis

**FIGURE 3:**
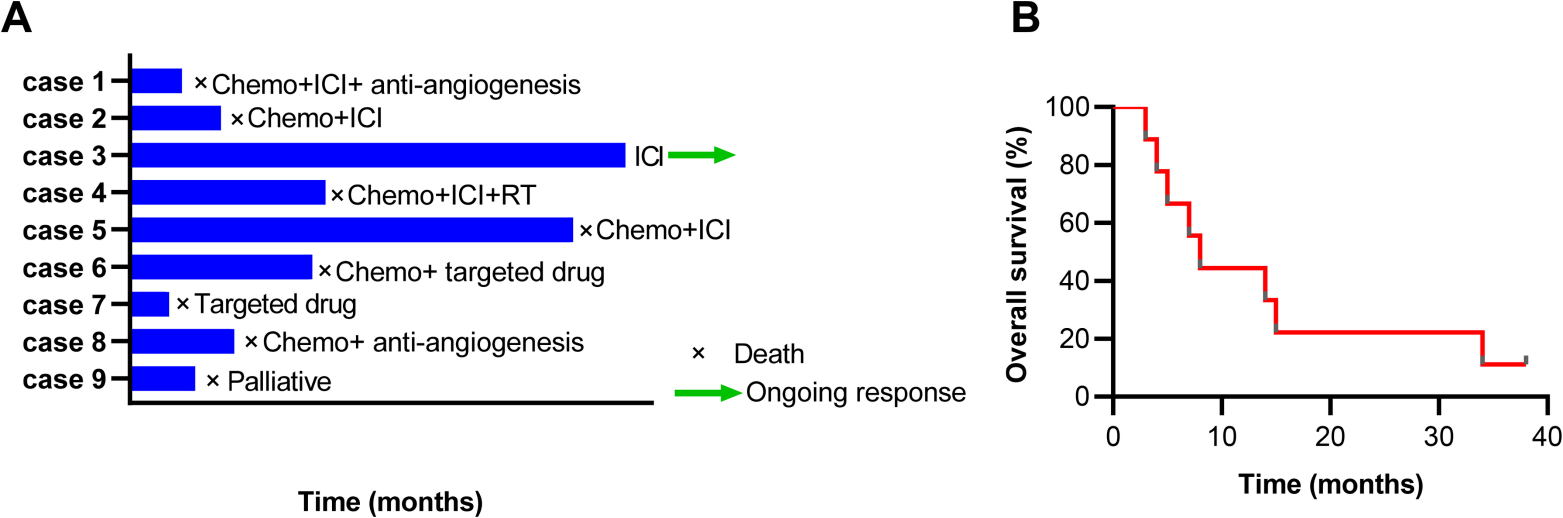
Summary of clinical information and therapies of all cases with thoracic SMARCA4‐UT. (A) Treatments details for all patients; symbols on each bar represent the treatment and prognosis status. (B) Overall survival curve of the patients analyzed using Kaplan–Meier curves. Chemo, chemotherapy; ICI, immune checkpoint inhibitors; RT, radiotherapy; SMARCA4‐UT, SMARCA4‐deficient undifferentiated tumors.

Eight cases received antitumor treatments (88.89%), with one opting for palliative care. Eight cases died with only one case still alive until our last follow‐up time of August 2025 (88.89%). The OS of one patient who did not receive antitumor treatment was 5 months. Chemotherapy was administered to six patients (66.67%), five of whom received ICI‐based therapy as first‐line treatment (tirellizumab, *n* = 1; atezolizumab, *n* = 1; triplimab, *n* = 1; carelizumab, *n* = 1; one patient enrolled in a clinical trial with the specific therapy undisclosed). In these cases, Case 1 experienced immunotherapy, chemotherapy and anti‐angiogenesis, but succumbed 4 months later. Case 2 harbored a KRAS gene mutation, was treated with anlotinib as third‐line antiangiogenic therapy, and experienced disease progression subsequent to a second‐line ICI‐based chemotherapy regimen. Despite 3 months of anlotinib treatment, the patient died 7 months after diagnosis. Case 4 received immunotherapy with chemotherapy as first‐line and subsequently radiotherapy, with OS of 15 months. Conversely, Case 5 received six cycles of immunotherapy and chemotherapy and then participated in a clinical trial administered with immunotherapy only, with OS of 34 months. Notably, of the five cases receiving immunotherapy, Case 3 consented to immunotherapy with triplimab alone, extended for 38 months, and he is still surviving at the latest follow‐up time of August 2025. All the above five patients receiving immunotherapy had a survival time ranging from 4 to 38 months, underscoring the significance of immunotherapy in this rare malignancy. Of the five cases that underwent genetic mutation analysis, three harbored gene mutations, with two cases administered with targeted therapy (22.22%). Case 6 declined treatment at our institution and sought treatment elsewhere, receiving chemotherapy and targeted therapy with limited details provided, with OS of 14 months. Another case, Case 7, had an ALK gene mutation with a PD‐L1 expression of 50% and was administered with an ALK inhibitor ensartinib as first‐line therapy due to a deteriorating general condition and refusal of chemotherapy with an OS of 3 months. There is only one case (Case 8) receiving chemotherapy in conjunction with antiangiogenesis treatments, with OS of 8 months. The detailed treatment regimens and outcomes were outlined in Table 3. A comprehensive summary of therapeutic disparities and Kaplan–Meier curves illustrating OS is depicted in Figure 3.

As comprehension of this rare tumor subtype advances, an increase in reported cases is anticipated, enriching our understanding of these malignancies. A comprehensive literature review was conducted to elucidate the treatment landscape and prognostic outcomes of thoracic SMARCA4‐UT patients. As depicted in Table 4, the majority were male patients with a significant smoking history. PD‐L1 expression varied widely, ranging from 0% to 100%. SMARCA4 exhibited different co‐mutations with TP53, KEAP1, ALK, and ROS. Chemotherapy‐based ICIs represent a crucial therapeutic approach for this tumor. Atezolizumab, pembrolizumab, tislelizumab, and nivolumab were the most commonly used ICIs; paclitaxel and carboplatin were frequently employed chemotherapy regimens, often followed by etoposide and carboplatin (EC). Notably, one case received PD‐1 blockade therapy in conjunction with anti‐CTLA‐4 therapies and survived for 26 months, highlighting the potential of dual immunotherapy combinations [14]. However, following the same treatment regimen, another case achieved a partial response for 3 months before succumbing, highlighting the possible risks associated with dual immunotherapies [15]. Our case series and literature review underscore the resistance to conventional treatments but potential benefits from immunotherapy of thoracic SMARCA4‐UT.

**TABLE 4:** Summary of reported cases of thoracic SMARCA4‐UT.

Case no.	Age (y)	Sex	Smoking (pack year)	PD‐L1%	Gene mutation	ICI	Chemo/RT/targeted drugs	Outcomes	Ref
1–10	48 (28–90)	M:F = 9:1	18.5 (5–60)	NA	SMARCA4 and TP53, complex genomic profiles	NA	Strategies diverse: chemotherapy, surgery, RT, EZH2 inhibitor	Median survival 6 months	[2]
11	41	M	Smoker	100	CDKN2A (p.T18fs), SMARCA4 (p.K1334fs)	Pembrolizumab, ipilimumab	PC and RT	26 months to death	[14]
12	73	M	50	10–20	No mutation	Ipilimumab, nivolumab	—	3 months to PR and died suddenly	[15]
13	50	M	36	> 90	NA	Tislelizumab	No	Seven cycles ICI, tumor reduction to 2022	[16]
14	36	M	Active smoker	45–50	NA	Pembrolizumab	PC	3 cycles to PR until January, 2023	[17]
15	73	F	53	40	SMARCA4 (c.1119‐1G>T), CHD2, TP53, ZFHX3, RET, RPTOR, ROS1, LRP1B, SPTA1, NF1NTRK3, LRP1B, REL	Atezolizumab	ABCP	Progressed after 17 months of PR to 2021	[18]
16	59	M	39	0	SMARCA4 (K755Nfs), TP53, ERBB3, TP53, SMARCA4, GATA2, PPP2R1A, MST1R, PAK5, ZFHX3, RNF43, GRM3, TSC2, EPHA3, EPHA3	Atezolizumab	ABCP	Progressed after 10 months of PR to 2021	[18]
17	64	F	44	80	SMARCA4 (H103Mfs), CREBBP, PLCG2, INSR, FLT4, KEAP1, ABL2, TSC2, TP53, SPTA1, LATS2, FAT1, ATM, EP300, STAT3	Atezolizumab	ABCP, RT	7 cycles to PR, maintenance therapy to 2021	[18]
18	62	F	40	25	ARID1A, CHEK2, KDM6, MUTH, SMARCA4, TP53	Pembrolizumab	EC	7 months to PR, alive 12 months to April, 2023	[19]
19	51	M	54	0	SMARCA4 (c.1105‐6G>T), TP53 (c.469‐5G>T), KEAP1 (p.T418fs)	Tislelizumab	First‐line: liposomal PC, anlotinib; second‐line: tislelizumab, EC	Second‐line 10 months to PR until June, 2022	[20]
20	76	M	49	< 1	SMARCA4 G1136fs, TP53H179R, and CDKN2A P81L	Nivolumab (third‐line)	First‐line: PC, RT; Second‐line: EC	Efficacy sustained 22 months until August, 2019	[21]
21	34	F	Never smoker	35	EMLA4 (exon 13), ALK (exon 20)	—	Targeted drug: alectinib	9 months to CR until December, 2022	[22]
22	51	M	22.5	0	SMARCA4 (L1161fs), TP53 (V157L)	Atezolizumab	ABCP	9 months PR after surgery to August, 2021	[23]
23	58	M	38	10	No mutation	Pembrolizumab	Pemetrexed, carboplatin	8 months to PR, survived 11 months to November, 2020	[24]

Abbreviations: ABCP, atezolizumab in combination with bevacizumab, paclitaxel, and carboplatin; Chemo, chemotherapy; CR, complete response; EC; etoposide and carboplatin; EZH2, enhancer of zeste homolog 2; F, female; ICI, immune checkpoint inhibitor; M, male; NA, not available; PC, paclitaxel and carboplatin; PD‐L1, programmed death ligand 1; PR, partial response; Ref, reference; RT, radiotherapy; SMARCA4‐UT, SMARCA4‐deficient undifferentiated tumors.

### 3.5 Genomic Analysis of SMARCA4 Mutations in Lung Cancer

**FIGURE 4:**
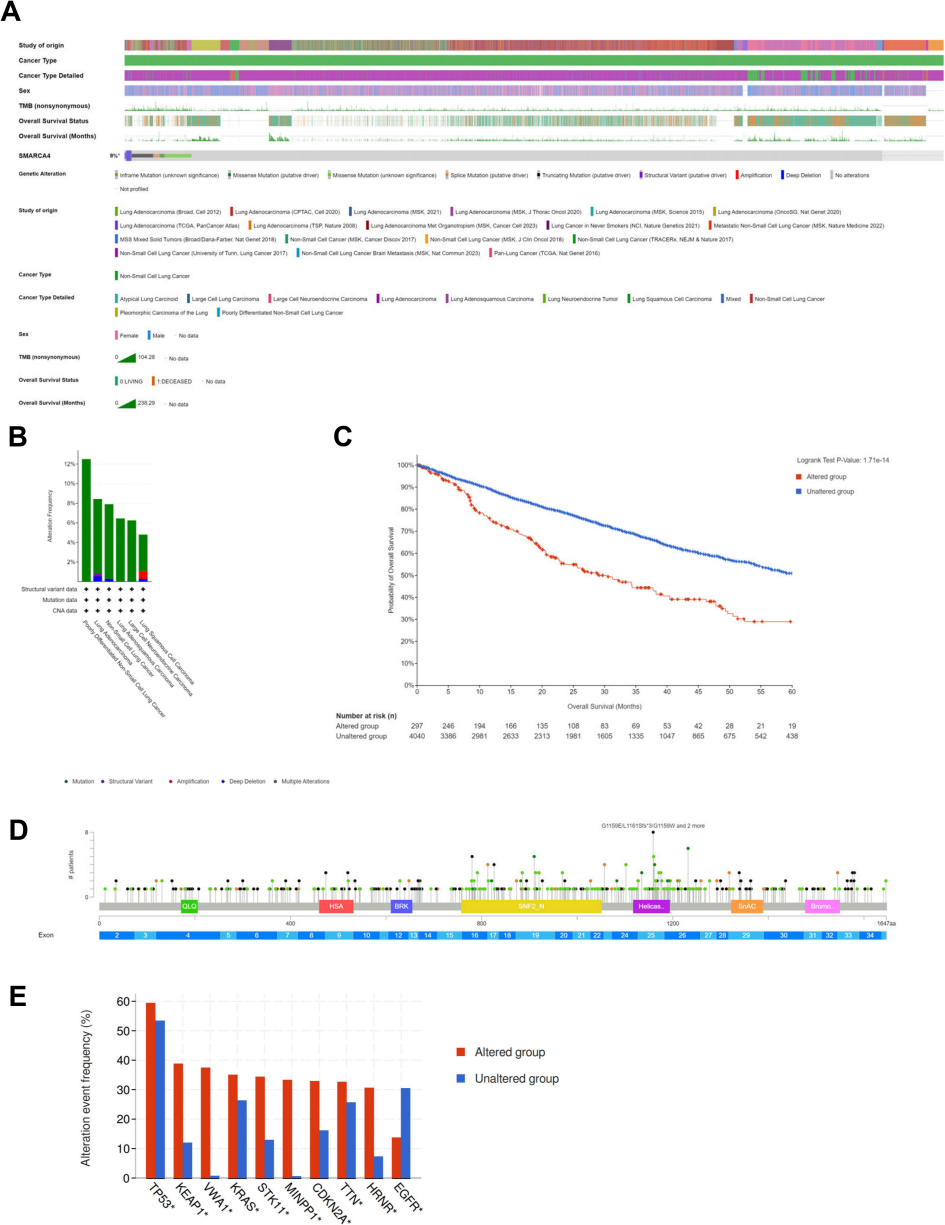
Genomic analysis of SMARCA4 mutations in NSCLC. (A) Oncoprint of SMARCA4 mutations found in patients with NSCLC across 22 studies. (B) Histograms illustrating the frequency of SMACA4 mutations in the NSCLC subset. (C) Kaplan–Meier curves showing overall survival in SMARCA4‐altered and unaltered groups of NSCLC patients. (D) Lollipop plot displaying the spectrum of SMARCA4 mutations with color‐coded motifs. Green QLQ, Gln, Leu, Gln motif; Red HSA, helicase/SANT‐associated domain; Blue BRK, Brahma and Kismet domain; Yellow SNF2_N, SNF2 family N‐terminal domain; Purple Helicas, conserved C‐terminal domain; Orange SnAC, Snf2‐ATP coupling, chromatin remodelling complex; Pink Bromo, bromodomain. (E) Frequency of co‐mutation genes in SMARCA4‐altered and unaltered NSCLC. NSCLC, nonsmall cell lung cancer.

The limited availability of pathological biopsy specimens precluded further genetic sequencing analysis in these patients. Consequently, we investigated the correlation between SMARCA4 mutations and disease progression in lung cancer patients, given the absence of genetic sequencing data for SMARCA4‐deficient undifferentiated thoracic tumors. This investigation utilized genetic sequencing data sourced from the cBioPortal database. Because of the scarcity of thoracic SMARCA4‐UT data in the database, we analyzed SMARCA4 mutations in lung cancer as a surrogate for this tumor type to some extent. Our analysis focused on genetic sequencing data from 8328 NSCLC patients across 22 studies, encompassing 10 609 samples, particularly emphasizing SMARCA4 gene mutations. The overall mutation frequency for SMARCA4 in NSCLC patients was 9% (Figure 4A). Poorly differentiated NSCLC subtypes exhibited the highest SMARCA4 mutation rate at approximately 12% (Figure 4B). Notably, NSCLC patients with SMARCA4 mutations displayed a 5‐year survival rate of 30%, which contrasted markedly with the approximately 60% survival rate of patients in the nonmutated group (Figure 4C). These findings underscore the prognostic significance of SMARCA4 mutations in NSCLC patients. SMARCA4 mutations were predominantly localized within the SNF2‐related domain and the helicase conserved C‐terminal domain (Figure 4D). The SNF2 protein, functioning as the ATPase component of the SNF2/SWI multisubunit complex, utilizes ATP hydrolysis‐derived energy to disrupt histone‐DNA interactions, thereby enhancing DNA accessibility to transcription factors. These structural domains likely play a pivotal role in the tumor‐suppressive function of SMARCA4, with postmutation effects significantly impacting patient prognosis. Additionally, SMARCA4 mutations frequently co‐occur with smoking‐related canonical mutations such as TP53, KRAS, and STK11 (Figure 4E).

In a subgroup of 239 small cell lung cancer (SCLC) patients across four studies, comprising 249 samples, the SMARCA4 mutation rate was only 2.9% (Figure S1A). Although the survival rate was relatively better than the unaltered group, only four patients exhibited significant mutations (Figure S1B). Further research is warranted to elucidate these findings.

## 4 Discussion

In 2017, SMARCA4‐deficient thoracic tumor was identified as a distinct entity characterized by aggressive behavior, rapid growth, early metastasis, and poor prognosis [3]. These tumors exhibit a greater tendency for invasion into surrounding tissues and distant organ spread at the time of diagnosis, leading to a worse prognosis compared to SMARCA4‐deficient NSCLC [10]. Primarily associated with smoking [12], these tumors have a reported median survival time of approximately 6 months [1, 4]. In our study, eight out of nine patients were male smokers, predominantly diagnosed at TNM stage IV. With a median OS of 8 months, our case series on SMARCA4‐UT contributes to some understanding of this rare malignancy.

The clinical presentation of SMARCA4‐UT lacks specific manifestations but commonly includes dyspnea, chest pain, weight loss, fatigue, superior vena cava syndromes, and pleural invasion [25]. In our cases, cough, sputum production, and chest pain were prevalent symptoms. These tumors often invade the mediastinum, pulmonary hilum, lung, pleura, cervical‐subclavian lymph nodes, forming large masses that compress surrounding tissues. Imaging studies typically reveal large, poorly demarcated masses in the mediastinum and lungs, with most cases diagnosed at an advanced stage. Imaging modalities such as CT and positron emission tomography/computed tomography (PET/CT) play a crucial role in detecting tumors and metastases [26]. Notably, our unique Case 3 presented with brain metastasis, a rare occurrence in SMARCA4‐UT [27]. There was only mention of one patient of SMARCA4‐UT by Sauter et al. that had a 1.4‐cm lung mass with brain metastasis [13]. Additionally, pulmonary vascular involvement [16] and diaphragmatic metastases, as observed in our patients, are uncommon findings. Case 3 presented involvement of the pulmonary vein and artery, and Case 2 has diaphragmatic metastases. To the best of our knowledge, this is the first case to report diaphragm invasion. The presence of lung emphysema was noted in a majority of patients with 50% showing this condition [27], and in our cases, 77.78% had emphysema. These findings further emphasize the need for considering SMARCA4‐UT in the differential diagnosis of thoracic lesions with compression of surrounding tissues, especially in young male heavy smokers.

Histologically, SMARCA4‐UT tumors are characterized by undifferentiated features, marked nuclear atypia, prominent nucleoli, pleomorphism, and rhabdoid cells, resembling malignant rhabdoid tumors [2, 3]. These tumors lack typical cellular differentiation markers observed in other NSCLCs and are negative for epithelial markers such as EMA and PCK [28]. Additionally, they do not stain positive for various keratins or markers of lymphocyte, melanocyte, myogenic, or vascular differentiation. For keratin including CK5/6 and CK7, our patients were negative or focally positive. Positive staining for SOX2, CD34, and SALL4, which are indicative of stem cell characteristics, was observed [29]. Notably, all patients were negative for TTF‐1, p40, and Napsin A. Importantly, preservation of SMARCB1 (INI‐1), another core regulatory subunit in the SWI/SNF complex, was consistent across all cases, aiding in the diagnosis of SMARCA4‐UT. Hence, SMARCA4‐UT should be strongly suspected in the poorly differentiated epithelioid tumors combined with negative SMARCA4/BRG1, TTF‐1, p40, Napsin A, and preserved SMARCB1 (INI‐1) results.

Current treatment strategies involve a combination of surgery, chemotherapy, immunotherapy, radiotherapy, and experimental targeted therapies [17]. Chemotherapy regimens commonly used for NSCLC have been employed in SMARCA4‐UT patients [18]. Some patients received first‐line EC strategies with a 7‐month duration to partial response (PR) [19] or second‐line treatment with a 10‐month duration to PR [20]. The most commonly used chemotherapy drugs in our cases were paclitaxel, carboplatin, gemcitabine, and platinum. Combination therapies, including pembrolizumab‐ipilimumab and ipilimumab‐nivolumab, have demonstrated positive outcomes, with prolonged OS of 26 months [14, 15]. In case of chemotherapy failure, nivolumab acted as a third‐line therapy in the context of negative PD‐L1 expression, resulting in a dramatic regression of tumor size to an almost undetectable level, with efficacy sustained for approximately 22 months [21]. Yang et al. reported a patient who was PD‐L1 negative was administered with tislelizumab‐based chemotherapy as a second‐line treatment, achieving more than 10‐month disease‐free survival [20]. Notably, immunotherapy showed promising effectiveness in our cases. Without PD‐L1 expression detection for Case 3, who was administered with triplimab only, had brain metastases and maintained survival for 38 months, and was alive until August 2025. Other four cases receiving ICI‐based therapy achieved OS ranging from 4 to 34 months, indicating the promising effectiveness of immunotherapy regardless of PD‐L1 expression. Moreover, the above cases of successful immunotherapy despite negative or low PD‐L1 expression in several patients are particularly noteworthy, demonstrating that PD‐L1 expression level may not be a reliable predictor of immunotherapy efficacy in thoracic SMARCA4‐UT. Therefore, patients with this type of tumor may potentially benefit from immunotherapy to some extent. Angiogenesis inhibitors like bevacizumab and anlotinib have also shown efficacy in combination with chemotherapy [18, 20]. In one case where bevacizumab and recombinant human endostatin were used, the patients died 4 months later. Moreover, Case 8 received chemotherapy combined with recombinant human endostatin and had an OS of 8 months. Although our study lacked sufficient cases for robust comparisons of treatment strategies, the use of immunotherapy in combination with chemotherapy might be a favorable approach for managing thoracic SMARCA4‐UT, regardless of PD‐L1 expression status.

Due to the absence of common driver gene mutations, it typically exhibits resistance to targeted drugs, and no specific targeted therapy has been approved for this tumor. Emerging targeted agents such as histone deacetylase inhibitors, enhancer of zeste homolog 2 (EZH2) inhibitors, cyclin inhibitors, and DNA damage repair inhibitors are being explored [9], with EZH2 inhibitors showing promising results [6]. Genetic testing in our cohort identified KRAS and ALK mutations in two male smokers, consistent with studies linking these mutations to smoking [10, 12]. A female patient with an ALK mutation responded well to alectinib, achieving PR after 1.5 months and a complete response after 9 months [22]. However, Case 7, who had an ALK mutation, received ensartinib and died 3 months later due to dyspnea and severe pulmonary infection. As far as we know, this is the second patient reported to receive an ALK inhibitor. Notably, it was reported by Rekhtman et al. that KRAS mutation occurs in 27.8% of SMARCA4‐UT patients [12]; the co‐occurrence of KRAS and SMARCA4 mutations was associated with a shorter OS and poor response to conventional treatments [30]. Case 2, who had a KRAS mutation but not a KRAS^G12C^, received ICI and chemotherapy. Unfortunately, the disease progressed and different treatment regimens were changed during treatment, leading to death 7 months later. Additional targeted therapies or personalized approaches may be considered in such cases.

The SMARCA4 gene encodes the BRG1 protein, a component of the SWI/SNF chromatin remodeling complex, and its loss disrupts chromatin remodeling activity, leading to widespread changes in gene expression patterns. The SNF2 protein, a component of the SNF2/SWI multisubunit complex, disrupts histone‐DNA interactions through ATP hydrolysis, thereby enhancing DNA accessibility to transcription factors [9]. These structural domains likely play a pivotal role in the tumor‐suppressive function of SMARCA4 and significantly impact the prognosis of NSCLC with post‐mutation. Structural variants, amplifications, and deep deletions as common forms of SMARCA4 inactivation were identified in our study. Mutations in SMARCA4 predominantly localize within the SNF2‐related domain and the conserved C‐terminal domain of the helicase, which could serve as crucial targets for future drug development. The overall mutation rate for SMARCA4 in NSCLC was 9% according to our genetic analysis, with poorly differentiated NSCLC exhibiting an approximately 12% mutation rate, highlighting the importance of vigilance regarding SMARCA4 mutations in lung cancer patients [12]. Co‐occurring mutations with smoking‐related canonical mutations, such as KRAS, STK11, and KEAP1, are common in patients with SMARCA4 mutations, and up to 44% of these patients exhibit a smoking‐related co‐mutation signature and a high tumor mutational burden [12, 28]. These findings suggest that SMARCA4 may have a similar effect on the prognosis of both NSCLC and thoracic SMARCA4‐UT, providing insights for future implications in prognosis and treatment strategies for SMARCA4‐deficient tumors.

Given the limited numbers, specific conclusions cannot be drawn. Assessing the efficacy of ICI, targeted therapies, or chemotherapy necessitates a large‐scale cohort study. Lack of comprehensive information due to patients seeking treatment at other institutions further complicates our analysis. Because of limited sample size, further genetic sequencing analysis could not be conducted. Considering the absence of genetic sequencing analysis for thoracic SMARCA4‐UT, we investigated the association between SMARCA4 mutations and disease progression in NSCLC and SCLC. Further investigation is warranted.

## 5 Conclusions

This study provides valuable insights into the clinical features, treatment outcomes, and genomic landscape of thoracic SMARCA4‐UT. Immunotherapy and targeted therapies offer hope for improved outcomes, with larger studies needed. Ongoing advancements and collaborative efforts are essential for developing novel therapies and improving outcomes for this challenging tumor.

## Author Contributions

F.Z.S. and N.N.Z. contributed to the study design, data interpretation, and manuscript drafting. S.T.L. conducted gene analysis. N.N.Z. and W.W. performed data collection and follow‐up. Z.T.S. oversaw imaging data. F.Z.S., Y.W.Z., and L.L.L. conducted literature reviews. B.D.L. was responsible for pathology. N.N.Z. and J.J.C. carried out statistical analyses. G.X.Y., X.M.Z., M.Y., and Y.M.M. assisted with data collection. N.N.Z. supervised the process and revised the manuscript. All authors have reviewed and approved the final manuscript for submission.

## Funding

This work was supported by the National Natural Science Foundation of China (grant number 82000090), the Natural Science Foundation of Shandong Province (Grant Number ZR2025MS1204), the Taishan Scholars Project of Shandong Province (Grant Number tsqn202507386), the Science and Technology Support Plan for Youth Innovation of Colleges and Universities of Shandong Province of China (Grant Number 2024KJJ004), and the High‐level Scientific Research Project Cultivation Project of Jining Medical University (Grant Number JYGC2022FKJ005).

## Ethics Statement

The study was approved by the ethics committee of the Affiliated Hospital of Jining Medical University (No. 2024‐03‐C021).

## Consent

Written informed consent was obtained from all patients or their next of kin.

## Conflicts of Interest

The authors declare no conflicts of interest.

## Supporting information


**Data S1:** Supporting Information.

**Figure S1:** Genetic sequencing analysis of SMARCA4 in SCLC.
(A) Oncoprint of SMARCA4 mutations found in patients with SCLC from 4 studies.
(B) Overall survival curve of SMARCA4‐altered and unaltered groups in SCLC. SCLC, small cell lung cancer.

## Data Availability

The data that support the findings of this study are available from the corresponding author upon reasonable request.
