# Supplementary material for: SMARCA4‐Deficient Undifferentiated Thoracic Tumor: Clinical Features and Prognosis of a Case Series and Literature Review
Source: Clin Respir J. 2026 Jan 23;20(1):e70168. doi: 10.1111/crj.70168 (PMC12830063; doi:10.1111/crj.70168)
Supplement: Supplementary file 1 — Data S1: Supporting Information. [file CRJ-20-e70168-s001.docx]

**Supplementary material**

**Supplementary Figure 1.** Genetic sequencing analysis of SMARCA4 in SCLC.

(A) Oncoprint of SMARCA4 mutations found in patients with SCLC from 4 studies. (B) Overall survival curve of SMARCA4-altered and unaltered groups in SCLC. Abbreviations: SCLC, small cell lung cancer.
