# Supplementary figures and images for: SMARCA4‐Deficient Undifferentiated Thoracic Tumor: Clinical Features and Prognosis of a Case Series and Literature Review
Source: Clin Respir J. 2026 Jan 23;20(1):e70168. doi: 10.1111/crj.70168 (PMC12830063; doi:10.1111/crj.70168)

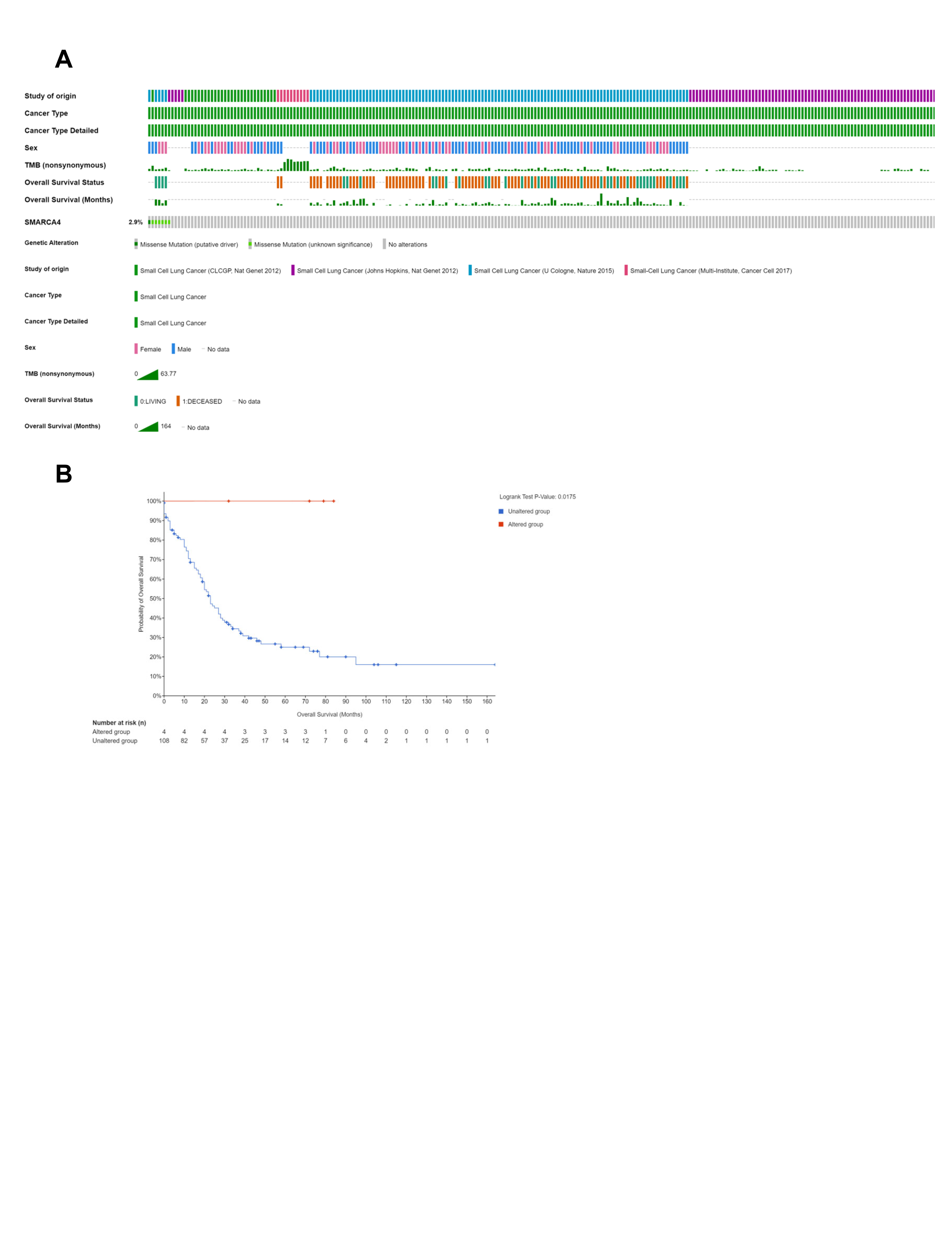

Supplement: Supplementary file 2 — Figure S1: Genetic sequencing analysis of SMARCA4 in SCLC. (A) Oncoprint of SMARCA4 mutations found in patients with SCLC from 4 studies. (B) Overall survival curve of SMARCA4‐altered and unaltered groups in SCLC. SCLC, small cell lung cancer. [file CRJ-20-e70168-s002.tif]
